# Atrioventricular re-entrant tachycardia and atrioventricular node re-entrant tachycardia in a patient with cancer under chemotherapy: a case report and literature review

**DOI:** 10.3389/fcvm.2024.1367893

**Published:** 2024-06-07

**Authors:** Meiyan Dai, Yue Chen, Jin Qin

**Affiliations:** ^1^ Division of Cardiology, Department of Internal Medicine, Tongji Hospital, Tongji Medical College, Huazhong University of Science and Technology, Wuhan, China; ^2^ Department and Institute of Infectious Disease, Tongji Hospital, Tongji Medical College, Huazhong University of Science and Technology, Wuhan, China

**Keywords:** chemotherapy, arrhythmia, cancer, ion channel, case report

## Abstract

Cardio-oncology is a new field of interest in cardiology focusing on the detection and treatment of cardiovascular diseases, such as arrhythmias, myocarditis, and heart failure, as side-effects of chemotherapy and radiotherapy. The association between chemotherapeutic agents and arrhythmias has previously been established. Atrial tachyarrhythmias, particularly atrial fibrillation, are most common, but ventricular arrhythmias, including those related to treatment-induced QT prolongation, and bradyarrhythmias can also occur. However, the association between chemotherapeutic agents and atrioventricular re-entrant tachycardia (AVRT)/atrioventricular node re-entrant tachycardia (AVNRT) remains poorly understood. Here, we report a patient with new-onset AVRT/AVNRT and lung cancer who underwent chemotherapy. We considered that chemotherapy or cancer itself may have been a trigger for the initiation of paroxysmal AVRT/AVNRT, and that radiofrequency catheter ablation was effective in treating this type of tachycardia. Here, possible mechanisms and potential genes (mostly ion channels) involved in AVRT/AVNRT are summarized and the mechanisms underlying the possible regulatory patterns of cancer cells and chemotherapy on ion channels are reviewed. Finally, we considered that ion channel abnormalities may link cancer or chemotherapy to the onset of AVRT/AVNRT. The aim of the present study was to highlight the association between chemotherapeutic agents and AVRT/AVNRT and to provide new insights for future research. Understanding the intermediate mechanisms between chemotherapeutic agents and AVRT/AVNRT may be beneficial in preventing chemotherapy-evoked AVRT/AVNRT (and/or other arrhythmias) in future.

## 1 Introduction

Cancer is a major disease threatening human health, accounting for nearly one in six deaths (1). Some cancers that have historically been associated with high fatality and mortality rates now have high cure rates, converting malignancy into a chronic disease (2). However, an increasing number of patients with cancer are at risk of the adverse effects of cancer therapy. Cancer therapy-related cardiac dysfunction is a serious side-effect of chemotherapy, occurring in approximately 10% of patients (3). Cancer therapy has been linked to myocyte damage, such as heart failure, thrombogenesis, pericardial pathology, hypertension, ischemia, vasospasm, and myocarditis (4–6). However, few reports have addressed cancer treatment-induced arrhythmia until recently.

Chemotherapeutic agents, such as anthracyclines, alkylating agents, antimetabolites, histone deacetylase (HDAC) inhibitors, immunomodulatory drugs, platinum compounds, proteasome inhibitors, multitargeted tyrosine kinase inhibitors, vascular endothelial growth factor (VEGF) signaling pathway inhibitors, and immune check point inhibitors, are suggested to be associated with a broad range of arrhythmias, including sinus bradycardia and tachycardia, premature atrial complexes, supraventricular tachycardia (SVT), premature ventricular complexes (PVCs), and ventricular tachycardia (VT) (2, 7–11). Accelerated apoptosis, inflammation, mitochondrial dysfunction, oxidative stress, and impairment of intracellular calcium (Ca^2+^) handling have been implicated in cancer therapy-induced arrhythmia (12–14). However, the detailed mechanisms through which chemotherapeutic agents cause arrhythmia remain unclear.

In terms of various arrhythmias, previous studies have reported the association between chemotherapeutic agents and SVT. SVT can be categorized as follows: atrioventricular re-entrant tachycardia (AVRT), atrioventricular node re-entrant tachycardia (AVNRT), atrial tachycardia, sinus tachycardia, sinus nodal re-entrant tachycardia, inappropriate sinus tachycardia, multifocal atrial tachycardia, junctional ectopic tachycardia, and non-paroxysmal junctional tachycardia. AVNRT and AVRT are types of paroxysmal supraventricular tachycardia (PSVT) that result from the presence of congenital re-entry circuits long before the development of cancer (15, 16). Currently, the association between cancer therapy and re-entrant tachycardia, such as AVNRT and/or AVRT, is poorly established.

Here, we report a case of new-onset AVRT/AVNRT in a patient with lung cancer who underwent chemotherapy. This case suggests that chemotherapy for cancer, or even the cancer itself, might be a possible trigger for the onset of AVRT/AVNRT. Moreover, as the direct mechanisms underlying cancer therapy-induced arrhythmia (especially AVNRT/AVRT) remain unclear, we summarize relevant evidence from available mechanistic studies. This study may provide insights into the development of new strategies and drugs to prevent and treat cancer therapy agent-induced arrhythmias.

## 2 Case report

### 2.1 Chemotherapy as a trigger for AVRT/AVNRT

**Figure 1:**
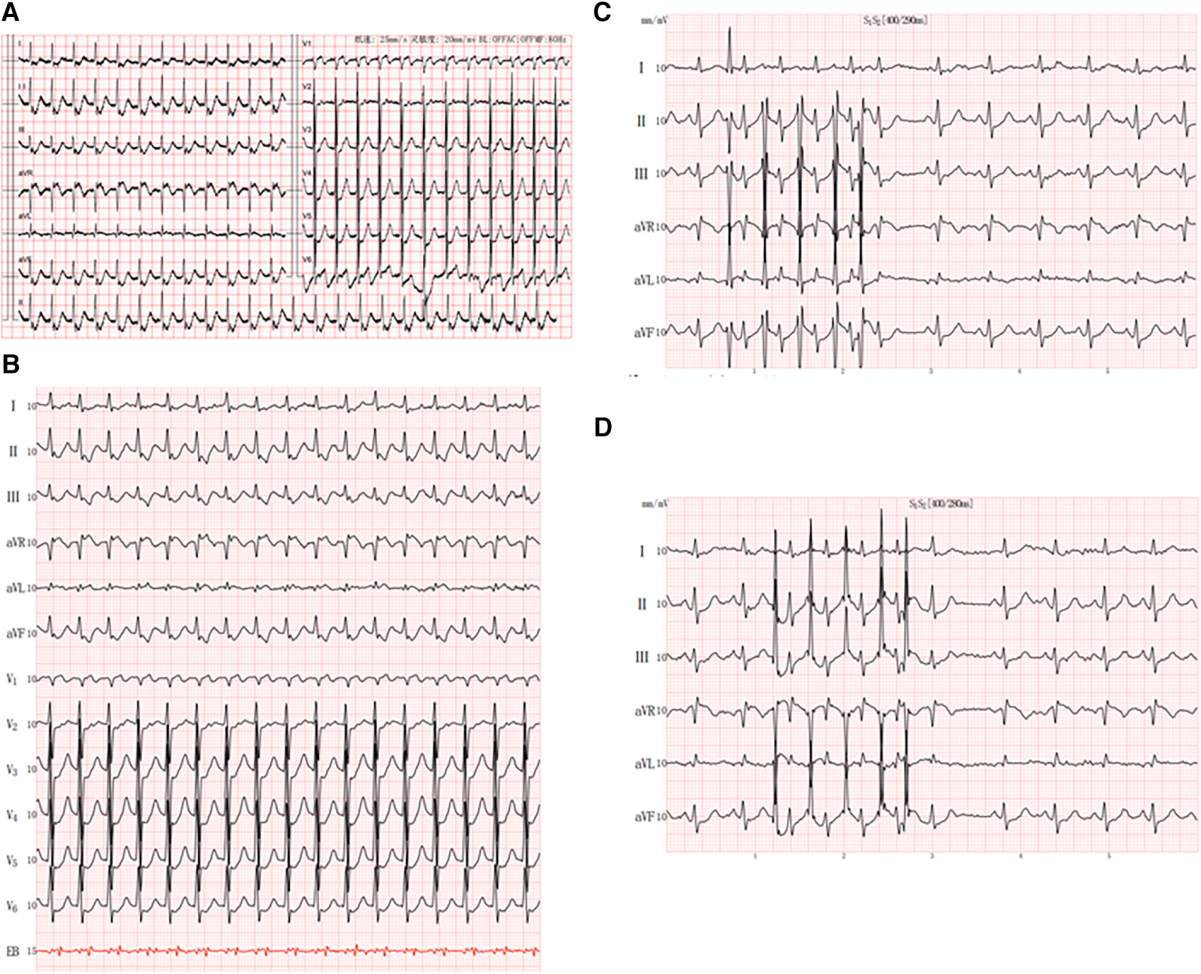
Patient characteristics: (**A**) 12-lead electrocardiogram showed a small-complex supraventricular tachycardia; (**B**) esophageal electrocardiogram showed RP’ < P’R and RP’ interval of 120 ms in V1; (**C,D**) jumping phenomenon caused by S_2_R stimulation.

**Figure 2:**
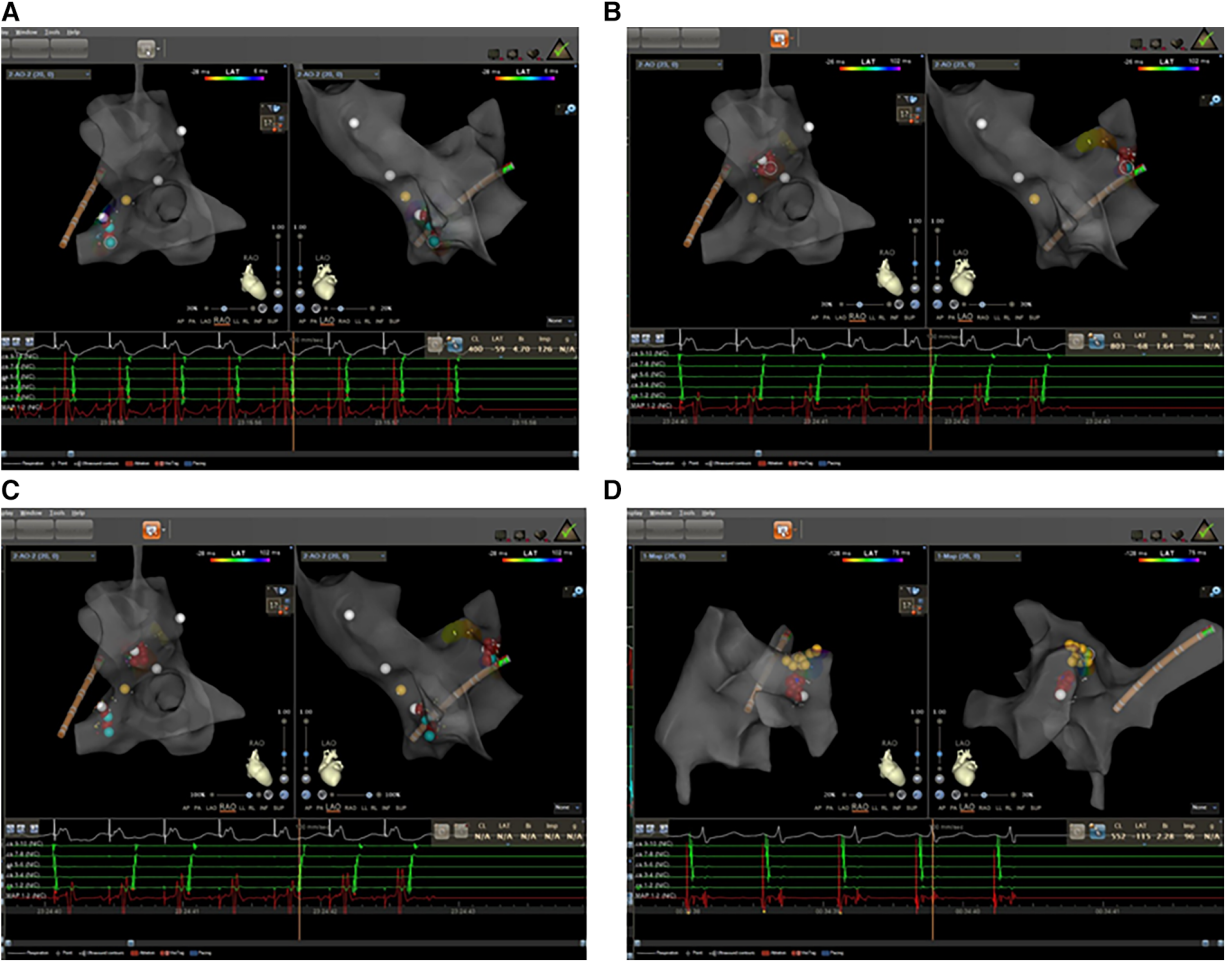
Electrophysiology study was performed with zero-fluoroscopy-approach-guided Carto 3TM system. (**A**) The earliest atrial activation was at CS7/8 with fixed retrograde atrial conduction under ventricular pacing. (**B**) The earliest atrial activation changed to CS1/2 with fixed retrograde atrial conduction under ventricular pacing. (**C**) The ablation targets of left lateral and septal accessory pathways. (**D**) The ablation targets of slow pathway modification. Red dots: ablation target; yellow dots: His bundle.

A 65-year-old man with a history of lung cancer and chemotherapy, admitted to hospital on 4 October 2023 with a 2-week history of right-sided chest pain, was diagnosed with lung adenocarcinoma (LUAD) (cT2N2MIaIVa). The patient denied a history of hypertension, diabetes, mental illness, or cardiovascular/cerebrovascular disease and did not receive any daily pharmacological treatment. The patient did not refer to any arrhythmia relevant to his medical history. On 12 October 2023, he was treated with pemetrexed and nedaplatin (NDP; intravenous injection) and endostatin (intrapleural infusion). On 30 October, he received a bevacizumab intravenous injection. During the course of the disease, the patient's serum electrolyte level was dynamically monitored, and the electrolyte balance was basically maintained; in particular, the serum potassium level was maintained at approximately 4.0–4.5 mmol/L. On 5 November, he complained of hourly palpitations and tachycardic episodes. A 12-lead electrocardiogram (ECG) showed a narrow QRS complex tachycardia (Figure 1A), which was refractory to intravenous therapy with propafenone and adenosine triphosphate. A subsequent transesophageal electrogram indicated a possible slow-fast AVNRT and orthodromic AVRT (Figures 1B–D). After obtaining patient and family consent, electrophysiology study and radiofrequency catheter ablation were performed using the Carto 3™ system. A decapolar catheter was advanced into the coronary sinus (CS) and a quadripolar catheter was positioned in the right ventricular apex. The patient was in normal sinus rhythm at baseline with proximal to distal coronary sinus activation. During the electrophysiology study, fixed retrograde atrial conduction was observed under ventricular pacing, with the earliest atrial activation at CS7/8. Coronary sinus pacing showed the jump-up phenomenon, and a narrow QRS tachycardia was stably induced. A combined analysis of electrophysiology study with transesophageal electrogram, a left posterior septal accessory pathway, and AVNRT were considered. The ablation catheter gained access to the left ventricle via a retrograde aortic approach, and the earliest atrial activation and local VA fusion was detected at CS7/8 under ventricular pacing (Figure 2A). After ablation with a 35 W discharge, the earliest atrial activation changed to CS1/2 with ventricular pacing (Figure 2B). Therefore, the patient appeared to have another concealed left lateral accessory pathway (Figure 2C). After ablation with a 35 W discharge, the local VA fusion was separated under ventricular pacing. Ventricular decremental pacing and ventricular extrastimuli did not demonstrate ventriculoatrial conduction anymore, while atrial extrastimulus testing demonstrated dual AV node physiology. During the coronary sinus stimulation with S1S2, the jumping phenomenon was observed, even though it could not induce tachycardia. After intravenous infusion of isoproterenol, coronary sinus stimulation revealed the jumping phenomenon and induced narrow QRS wave tachycardia stably. During tachycardia, the retrograde A wave in the coronary sinus electrode remained in a straight line, leading to the diagnosis of slow-fast atrioventricular nodal re-entrant tachycardia. A slow pathway ablation was performed (Figure 2D), eliminating the dual AV node physiology even with programed atrial stimulation with and without isoproterenol. The patient reported no palpitations during a follow-up phone call 4 months after the procedure.

### 2.2 Genes/pathways associated with AVRT/AVNRT

AVNRT occurs when a re-entry circuit is formed within or in close proximity to an AV node (17). Dual AV nodal conduction is a congenital abnormality that develops during prenatal cardiogenesis (18). AVRT is a macro-re-entrant tachycardia with an anatomically congenital circuit consisting of two distinct pathways (19). While these are congenital abnormalities, some studies have reported that the onset of AVNRT/AVRT can be triggered through several factors.

AVNRT is twice as common in women as in men and is the most common arrhythmia encountered during pregnancy, with approximately 44% of patients with known AVNRT experiencing symptoms during pregnancy (20, 21). Women with AVNRT are known to be significantly younger at symptom onset than men (22); therefore, sex hormones may also trigger tachycardia.

It has been suggested that AVNRT is an ion channel disease. Familial clustering indicates the involvement of genetic factors in AVNRT pathophysiology. Andreasen et al. reported that 26.4% of patients with AVNRT had ≥1 variant in genes affecting sodium handling, namely, *SCN3A*, *SCN5A*, *SCN10A*, *SCN8A*, *SCN4A*, *SCN1A*, *SCN2B*, and *SCN9A*, and 19.0% of patients with AVNRT had variants in genes affecting calcium handling (*RYR2*, *RYR3*, *CACNB2*, *ATP2A2*, *CACNA1C*, *CACNA1D*, *CACNA1I*, and *CACNA1G*) in the heart. Moreover, variants in *HCN1–4* and *KCNE3* have also been associated with AVNRT (23).

One study performed whole-exome sequencing (WES) in 82 patients with AVNRT and in 100 controls. They reported that the genes *SCN1A*, *PRKAG2*, *RYR2*, *CFTR*, *NOS1*, *PIK3CB*, *GAD2*, and *HIP1R* and the related pathways mediated by these genes, such as neuronal system/neurotransmitter release cycles or ion channels/cardiac conduction, might be involved in AVNRT (24, 25).

In terms of another type of PSVT, AVRT had a similar incidence in both young and older adults. The male/female ratio was similar across all age ranges (26). Familial Wolff–Parkinson–White syndrome (WPW), which is a common cause of AVRT, is well recognized as a cardiovascular disease, partly due to mutations in genes such as *PRKAG2* (27, 28). Pathogenic variants of MYH7 and rare *de novo* variants of genes associated with arrhythmia and cardiomyopathy (*ANK2*, *NEBL*, *PITX2*, and *PRDM16*) have been suggested as the genetic basis of WPW (29).

**Figure 3:**
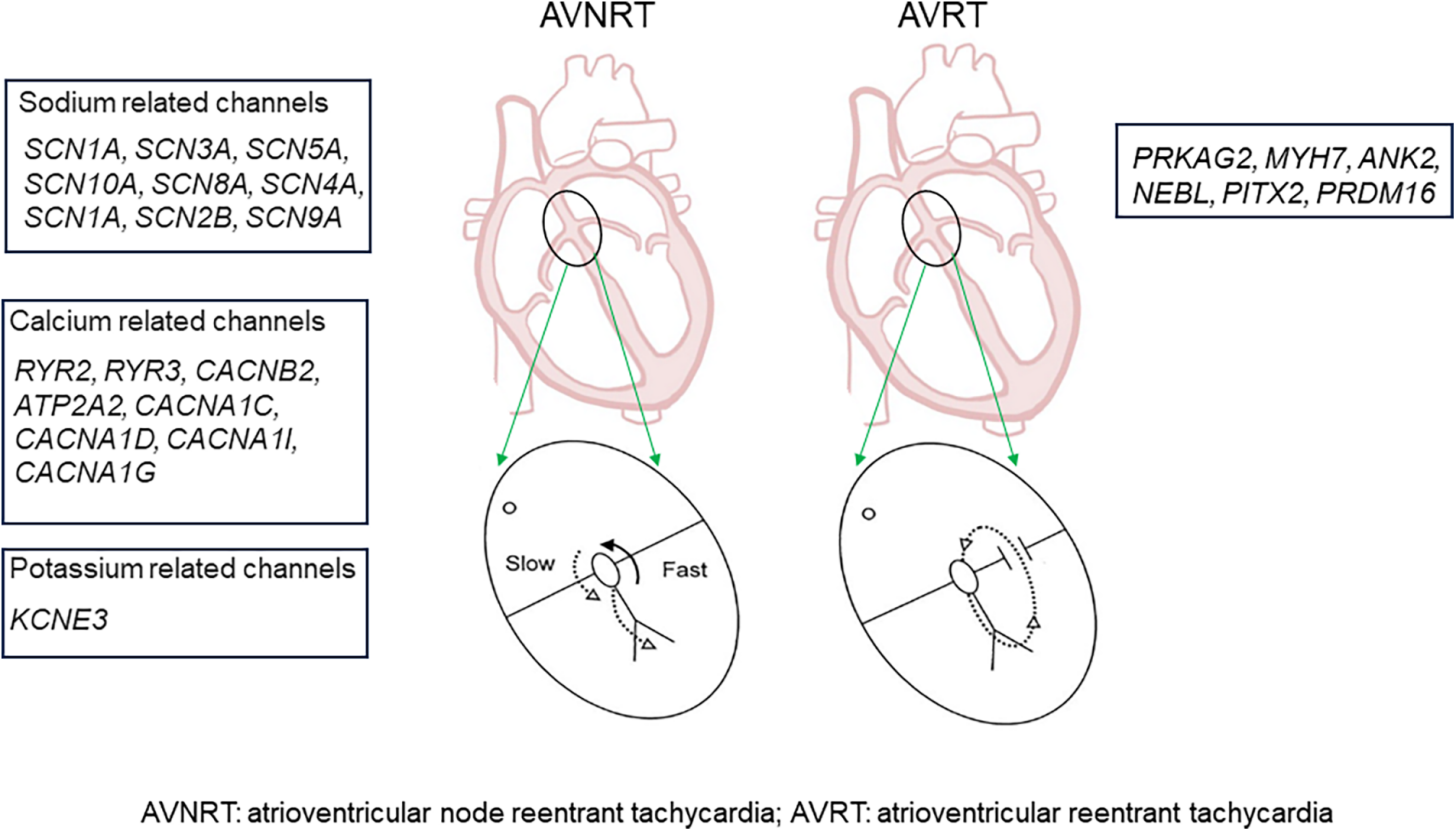
Genes that are potentially associated with AVNRT and AVRT.

Therefore, compared with AVRT, AVNRT appears to be more intricately linked to ion channels if not caused by ion channel dysfunction, and the onset of symptoms is largely influenced by ion channels (Figure 3).

### 2.3 Regulation of ion channels in cancers

Previous studies have reported the dysregulation of ion channels in cancer cells. One study reported that the voltage-gated K^+^ channel of Kv10.1 (EAG1) was aberrantly expressed in human pancreatic ductal adenocarcinoma (30). Moreover, EAG1 channels have been detected in approximately 70% of tumor biopsies originating from various cancers (31). Another K^+^ channel, KCNQ1 (potassium voltage-gated channel subfamily Q Member 1), is also associated with the development of colorectal cancer (CRC) (32). In breast and prostate cancers, decreased expression of the K^+^ channel KCNA3 has been found to be associated with an increased tumor grade. The K^+^ channel HERG1 (KCNH2) is upregulated in CRCs. The K^+^ channels EAG2 (KCNH5) and KCNT2 are highly expressed in the medulloblastoma of pediatric brain tumors. KCNA5, KCNQ1, and KCNN4 are also abnormally expressed in certain cancers (33).

Na^+^ channels include the voltage-gated sodium channel (VGSC) and ligand-gated sodium channel (LGSC) subfamilies. VGSC contains nine subtypes of Nav1.1–Nav1.9, including both α and β subunits. The expression of Nav1.5, Nav1.6, and Nav1.7 is upregulated in many cancer types, such as prostate, breast, lung, and cervical cancer, and leukemia (34).

Dysregulation of Ca2+ channels has also been shown to be involved in the development of various types of cancer (35, 36). For example, in breast cancer, prostate cancer, and leukemia, the voltage-gated Ca2+ channels are upregulated (37). Notably, ion channel-related proteins in cancer cells are secreted by small extracellular vesicles (sEVs). Proteomic profiling of small extracellular vesicles secreted by human pancreatic cancer cells has shown the presence of KCNN4, KCNK5, and KCNMA1 in extracellular vesicles (38). KCNN4 has also been reported to be detectable in cancer extracellular vesicles in breast, colon, and melanoma cancers. KCNAB3 is expressed in the extracellular vesicles of ovarian cancer cells. KCNQ1 and KCNH8 are present in the EVs of colon cancer. KCNAB2 is measurable in extracellular vesicles in breast, colon, and kidney cancers, and in leukemia and melanoma. KCNH4 has been detected in the extracellular vesicles of breast and lung cancer cells. The Ca2+ channel CACNA2D2 has been detected in breast, colon, melanoma, and ovarian extracellular vesicles. CACNA2D1 is highly expressed in breast, brain, lung, melanoma, and prostate cancer extracellular vesicles. CACNA1E has been detected in the extracellular vesicles of the breast, brain, colon, kidney, and ovaries (39, 40). sEVs can efficiently translocate to the cardiomyocytes (41, 42). Mechanistically, cancer cell-derived sEVs carrying aberrant ion channels that translocate into the heart may mediate ion homeostasis between cancer cells and cardiomyocytes.

Ion homeostasis affects cancer apoptosis and cell migration, and regulates the release of neurotransmitters, hormones, and growth factors in both normal cells (such as myocytes) and neoplastic cells (43, 44). Cancer cell secretions appear to indirectly induce ion channel dysfunction in myocytes. After treatment with cancer cell secretions, the maximum depolarization velocity and action potential amplitude in human-induced pluripotent stem cell (iPSC)-derived cardiomyocytes were reduced. Moreover, the action potential duration was prolonged, the peak Na^+^ and transient outward currents decreased, and late Na^+^ and slowly activating delayed rectifier K^+^ currents increased. Mechanistically, DNA methylation of ion channel genes via activation of TGF-β/PI3K signaling might contribute to ion channel dysfunction (45).

Moreover, cancer cells metabolize glucose via glycolysis, and hypoxia can further aggravate dependence on glycolytic fueling, resulting in the overproduction of lactic acid (46, 47). Elevated circulating lactate levels have been documented in patients with various types of cancers (breast, prostate, colorectal, lung, and ovarian) (48–51). Lactate potently regulates ion channels in the muscle cells. For example, Ca^2+^-activated K^+^ channels (KCa channels) are activated by lactate in smooth muscle cells. Keung and Li reported that lactate activates KATP channels in guinea pig myocytes, and Han et al. reported that lactate induces the opening of KATP channels in rabbit ventricular myocytes (52, 53). Lactate has also been shown to modify the fast sodium current through hyperpolarization in guinea pig myocytes (54), which may contribute to the development of arrhythmia.

Investigators have found that tumor cells secrete neurotrophic growth factors, axon guidance molecules, VEGF, and chemical messengers (55–57). Many cancer cells synthesize and release catecholamines. Norepinephrine levels are higher in pancreatic cancer tissue than in normal tissue (58). Plasma norepinephrine and epinephrine concentrations are significantly upregulated in patients with oral and oropharyngeal squamous cell carcinoma (SCC) compared with those in patients with no cancer (59). Plasma-free metanephrine and normetanephrine levels are elevated in patients with gastric carcinoma and lung cancer (60, 61). Catecholamines play major roles in the induction of cardiac ion channel dysfunction and rhythm disorders (62).

**Figure 4:**
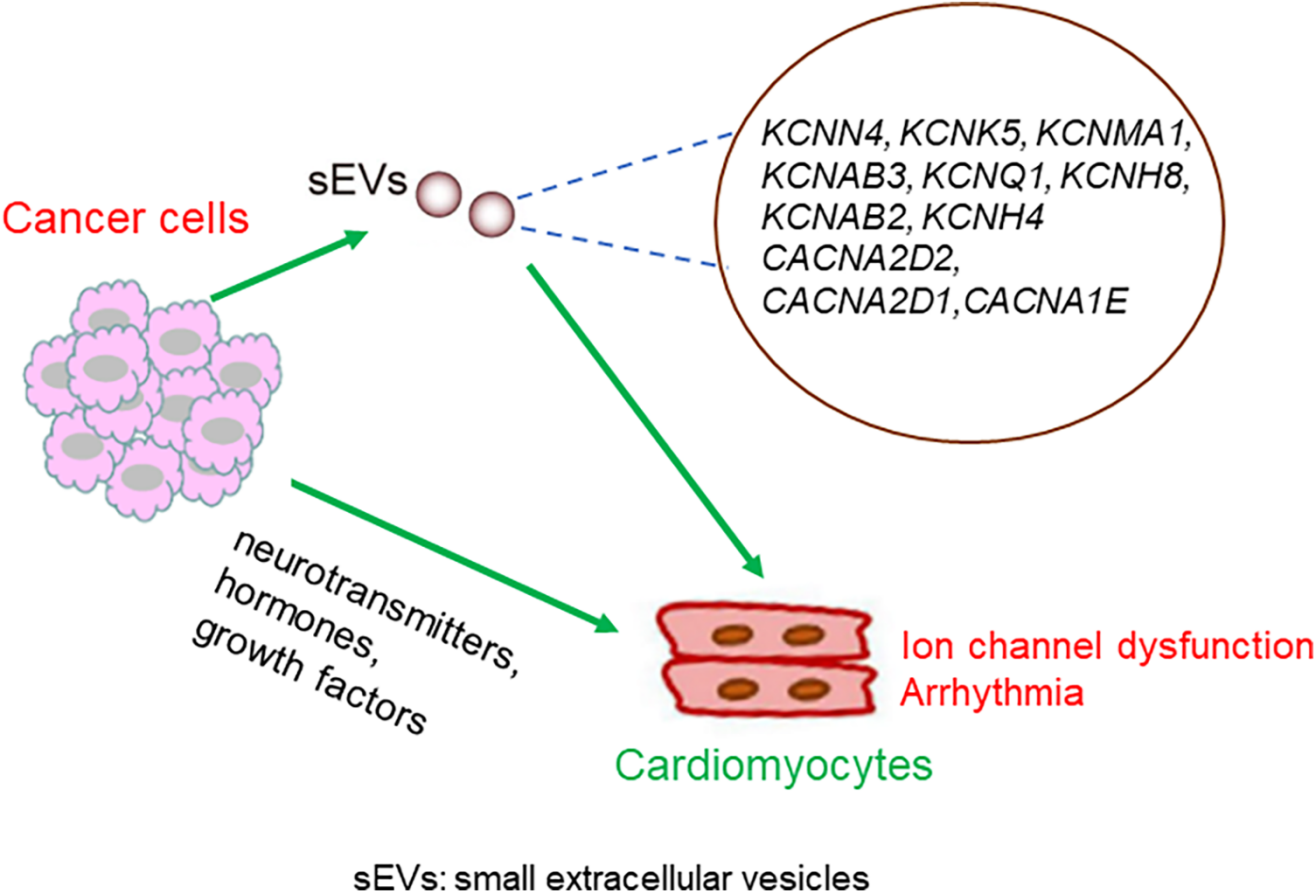
Possible mechanisms underlying the crosstalk between cancer cells and cardiomyocytes. Molecules secreted from cancer cells translocate into the heart, either through sEVs or other forms of particles, and regulate the expression or activity of cardiomyocytes localized ion channels. Alternatively, neurotransmitters, hormones, and growth factors secreted from cancer cells might mediate the crosstalk between cancer cells and cardiomyocytes.

The possible mechanisms linking cancer cells and the dysregulation of myocyte-localized ion channels are summarized in Figure 4.

### 2.4 Regulation of ion channels under chemotherapy

Ion channels are key regulators of cancer cell pathophysiology (63). Anticancer therapeutics appear to play non-negligible roles in the regulation of ion homeostasis in tumor cells.

Zhang et al. reported that paclitaxel, considered the most significant advance in chemotherapy in the past two decades, accelerates Ca^2+^ oscillations through increasing the IP3R opening frequency (64). Kang et al. showed that trifluoperazine, a well-known antipsychotic drug with anticancer effects, suppresses glioblastoma invasion through binding to the Ca^2+^-binding protein calmodulin subtype 2 (CaM2) (65).

The chemotherapeutic drug cisplatin activates the K^+^ channel KCa3.1, with the activation effect most likely due to the increase in intracellular Ca^2+^ concentration (66). Reduction in the activity of KCa3.1 has also been demonstrated with oxaliplatin treatment (67).

In non-tumor tissues/cells, chemotherapy can induce ion channel dysfunction. The development of acute oxaliplatin-induced peripheral neuropathy has been suggested to be due to changes in voltage-gated sodium channels, voltage-gated calcium channels, and in the TREK-1 two-pore-domain background K^+^ channel (68–70). Chemotherapy directly alters the ion channel activities of cardiomyocytes. Chemotherapy drugs and HDAC inhibitors have been associated with QTc prolongation and arrhythmias, and blockade of the KCNH2 by HDAC inhibitors may be a mechanistic explanation (71). Ibrutinib upregulates calmodulin kinase 2 expression and increases the phosphorylation of ryanodine receptor 2 (RyR) in the cardiomyocyte endoplasmic reticulum, impairing intracellular calcium handling and triggering ectopic electrical activity (72). PD-1 deficiency results in premature mortality due to high-titer IgG autoantibodies against cardiac troponin I, augmenting the voltage-dependent L-type calcium current in normal cardiomyocytes (73). Anti-HER2 therapy also causes calcium homeostasis dysfunction within cardiomyocytes, leading to Reactive Oxygen Species (ROS) activation within the myocardium, similar to that caused by anthracyclines or other antineoplastic agents (74). Calcium oscillations in cardiac cells that alter automaticity have been proposed to be the mechanism underlying Bruton kinase inhibitor-induced cardiac toxicity (75).

**Figure 5:**
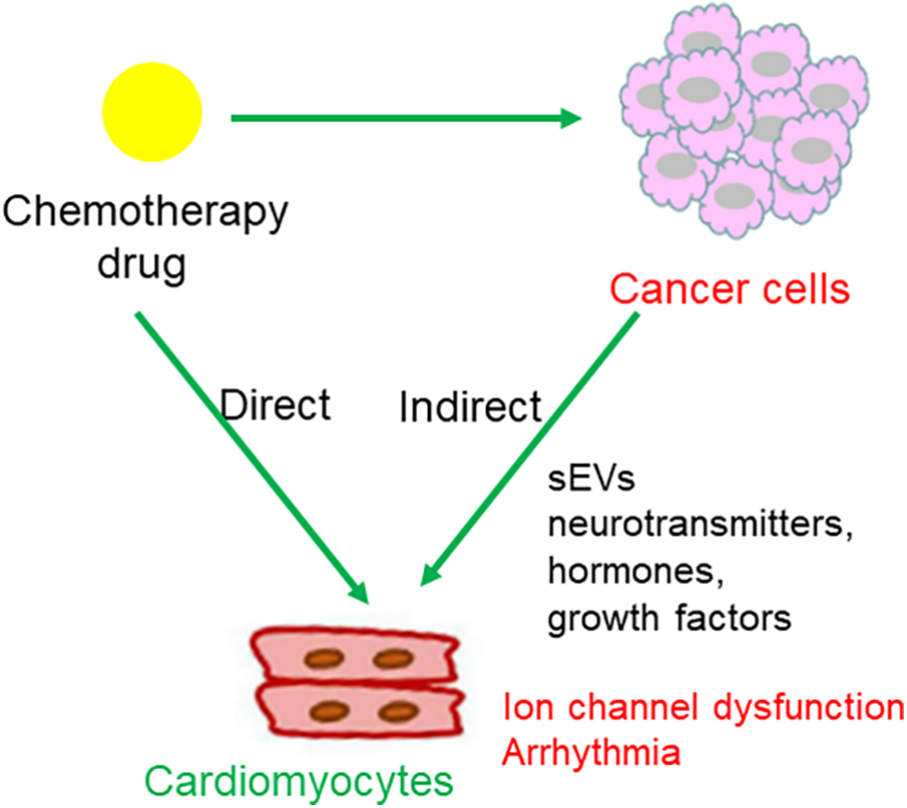
Regulatory pattern of chemotherapy drugs on cardiomyocytes. (1) Chemotherapeutic agents act upon cancer cells, leading to cancer cell death and subsequent release of chemical messages to indirectly influence cardiomyocytes localized ion channels; (2) direct action of chemotherapeutic agents on ion channels in cardiomyocytes.

The possible mechanisms linking chemotherapy with dysfunction of myocyte-localized ion channels are summarized in Figure 5.

### 2.5 Chemotherapy associated arrhythmias in LUAD

It has been suggested that chemotherapeutic agents are associated with a variety of arrhythmias. Combination chemotherapy of pemetrexed and carboplatin is a standard treatment approach for non-small cell lung cancer (NSCLC). A case report showed pemetrexed and carboplatin appear to have triggered sinus arrhythmia in a patient undergoing multiple courses of chemotherapy (76). However, the mechanisms underlying the case of sinus arrhythmia are unclear. NDP is a second-generation platinum derivative, which has similar antitumor activity to cisplatin with less nephrotoxicity and gastrointestinal toxicity. NDP-induced cardiotoxicity is rare, although it has been presented that three patients who were treated with NDP developed chemotherapy-induced serious arrhythmias. The three cases developed sinus tachycardia and atrial premature beats, complete left bundle branch block, and bigeminy ventricular premature contraction, in the second, sixth, and second cycles, respectively (77). The arrhythmias in these patients were resolved after drug treatment, withdrawal of chemotherapy, or adjustment of chemotherapy regimens.

Treatment with bevacizumab increases the risk of arterial adverse events, particularly cardiac and cerebral ischemia, venous adverse events, bleeding, and arterial hypertension (78). However, bevacizumab-associated arrhythmia is also rare. One case reported a patient with lung adenocarcinoma who was treated with bevacizumab monotherapy, and a poor R-wave increase with slight ST segment elevation in V1-V3 leads and ventricular arrhythmia were detected. The patient’s chest tightness and rapid heartbeat disappeared after the amiodarone treatment. The ECG monitoring results returned to normal (79).

It has been hypothesized that chemotherapeutic drug-associated cardiotoxicity occurs due to electrolyte imbalance or disturbance of the sinoatrial node. Herein, it is important to note that our patient developed ANRT and AVNRT during the first course of chemotherapy, without any electrolyte imbalance. However, it is important to consider that multiple factors, including chemotherapeutic drug combination, the selective cardiotoxicity of this chemotherapeutic regimen, aging, and heart disease-related risk factors, are all likely to have contributed to our patient’s situation.

KCNQ1 is implicated in long QT syndrome (LQTS) and cardiac arrhythmia; the clinical significance and biological role of KCNQ1 in LUAD is also described. The potential mechanism of KCNQ1 underlying LUAD progression may lie in the perturbation of genes relevant to the cell cycle and DNA replication. However, KCNQ1-inhibitor gefitinib, which is the first-generation targeted therapy for non-small cell lung cancer, was implicated in the induction of heart QT prolongation in a guinea pig model, thereby raising a concern of arrhythmia when gefitinib is used for NSCLC treatment (80).

## 3 Conclusions and perspectives

An estimated 32%–40% of the population has dual atrioventricular nodal physiology; however, only a minority develop AVNRT (81). The incidence of AVNRT is 35 per 10,000 person-years or 2.29 per 1,000 persons, and it is the most common form of non-sinus tachydysrhythmia in young adults (82). Regarding AVRT, one study reported a tachyarrhythmia rate of 1.0% per year in individuals with a WPW pattern (83). Therefore, the onset of AVNRT or AVRT in patients with dual atrioventricular nodal pathways or accessory atrioventricular pathways is rare, and when they occur, they are usually attributed to stress in relation to pregnancy, exertion, caffeine, alcohol, beta-agonists (salbutamol), or sympathomimetics (amphetamines) (84, 85). Chemotherapeutic agents directly promote various arrhythmias, such as atrial fibrillation (AF) and ventricular ectopic beats. However, chemotherapy-induced AVRT/AVNRT has rarely been reported. Here, we present a typical case of chemotherapy-related AVRT/AVNRT. Moreover, we propose the following potential mechanisms underlying chemotherapeutic agent-induced AVRT/AVNRT: (1) molecules secreted from cancer cells translocate into the heart, either through sEVs or other forms of particles, regulating the expression or activity of cardiomyocyte-localized ion channels; (2) chemotherapeutic agents act on cancer cells, leading to cancer cell death and the subsequent release of chemical messages to indirectly influence cardiomyocyte-localized ion channels; and (3) direct action of chemotherapeutic agents on ion channels in cardiomyocytes.

Animal models are of vital importance for investigating the cause-effect relationship and detailed mechanisms between chemotherapy and arrhythmias, such as AVRT/AVNRT. Mice are the primary animal models for studying arrhythmias, particularly genetic arrhythmia syndromes. *RYR2* mutations have been found to be implicated in several arrhythmia disorders, including catecholaminergic polymorphic ventricular tachycardia (CPVT), PVC ventricular fibrillation, and AF (86). KCNQ1 or CACNA1C (Cav1.2) overexpression has been associated with LQTS, sympathetic stimulation-induced early after depolarizations (EADs), and VT; mice with HCN4 (HCN4) ablation exhibited bradycardia, exit block, SVT, VT, and complete block (86). However, mouse models of AVRT/AVNRT have not been well established, partly because of technical difficulties in performing esophageal electrophysiological examinations or intracardiac electrophysiological studies on mice. Therefore, in the absence of an effective animal model, it is difficult to determine the exact pathophysiological basis of AVNRT. Further studies are required to establish animal models and develop electrophysiological examination technologies for small animals (such as mice). For *in vitro* studies, patient-specific iPSC-derived cardiomyocytes and human iPSC-derived 3D organotypic cardiac microtissues might be attractive experimental platforms to investigate the regulation of secretions from cancer cells and chemotherapeutic agents on ion channel activities on a large scale (87, 88). Moreover, high-throughput sequencing and mass spectrometry methods would allow for the systemic investigation of the regulation of ion channels by different types of chemotherapeutic agents. Further studies are required to identify the chemotherapeutic agents that are strongly associated with ion channel dysfunction. In clinical practice, agents that do not cause ion channel dysfunction are preferred.

Mechanistically, the direct regulation of cardiomyocytes by chemotherapeutic agents and the indirect regulation of cancer cells (with or without chemotherapy) on cardiomyocytes, such as sEV-mediated cancer-myocyte crosstalk, should be thoroughly investigated. Cell co-culture and Transwell experiments are helpful for studying complicated cancer-myocyte crosstalk.

In terms of treatment, arrhythmias are considered one of the clinical manifestations of heart failure and cardiomyopathy. Permanent treatment, such as ablation therapy, should be considered for arrhythmias with high success or cure rates, such as atrial flutter, AVNRT, AVRT, and atrial tachycardia (89). In this case, SVT was refractory to intravenous therapy with propafenone and reappeared repeatedly until ablation therapy successfully prevented the onset of AVNRT/AVRT after chemotherapy. Therefore, radiofrequency ablation may be an option for patients with arrhythmias after chemotherapy and poor response to antiarrhythmic drugs.

## Data Availability

The original contributions presented in the study are included in the article/Supplementary Material, further inquiries can be directed to the corresponding author.
